# The Neural Bases of Language Processing During Social and Non-Social Contexts: A fNIRS Study of Autistic and Neurotypical Preschool-Aged Children

**DOI:** 10.21203/rs.3.rs-4450882/v1

**Published:** 2024-06-06

**Authors:** Meredith Pecukonis, Javier Gerson, Hailey Gustafson-Alm, Maegan Wood, Meryem Yücel, David Boas, Helen Tager-Flusberg

**Affiliations:** Boston University; Boston University; Boston University; Boston University; Boston University; Boston University; Boston University

**Keywords:** autism, preschool, fNIRS, language, live, social context

## Abstract

**Background:**

Little is known about how the brains of autistic children process language during real-world “social contexts,” despite the fact that challenges with language, communication, and social interaction are core features of Autism Spectrum Disorder (ASD).

**Methods:**

We investigated the neural bases of language processing during social and non-social contexts in a sample of *N*=20 autistic and *N*=20 neurotypical (NT) preschool-aged children, 3 to 6 years old. Functional near-infrared spectroscopy (fNIRS) was used to measure children’s brain response to “live language” spoken by a live experimenter during an in-person social context (i.e., book reading), and “recorded language” played via an audio recording during a non-social context (i.e., screen time). We examined within-group and between-group differences in the strength and localization of brain response to live language and recorded language, as well as correlations between children’s brain response and language skills measured by the Preschool Language Scales.

**Results:**

In the NT group, brain response to live language was greater than brain response to recorded language in the right temporal parietal junction (TPJ). In the ASD group, the strength of brain response did not differ between conditions. The ASD group showed greater brain response to recorded language than the NT group in the right inferior and middle frontal gyrus (IMFG). Across groups, children’s language skills were negatively associated with brain response to recorded language in the right IMFG, suggesting that processing recorded language required more cognitive effort for children with lower language skills. Children’s language skills were also positively associated with the difference in brain response between conditions in the right TPJ, demonstrating that children who showed a greater difference in brain response to live language versus recorded language had higher language skills.

**Limitations:**

Findings should be considered preliminary until they are replicated in a larger sample.

**Conclusions:**

Findings suggest that the brains of NT children, but not autistic children, process language differently during social and non-social contexts. Individual differences in how the brain processes language during social and non-social contexts may help to explain why language skills are so variable across children with and without autism.

## Background

Language skills vary greatly across children with Autism Spectrum Disorder (ASD). While some autistic children go on to develop spoken language skills that are similar to, or even surpass, their neurotypical (NT) peers, many continue to have challenges using and/or understanding spoken language as they enter school-age (Bennett et al., 2014; Boucher, 2012; DiStefano & Kasari, 2016; Wodka, Mathy, & Kalb, 2013). Neurobiological factors, such as individual differences in how the brain processes language, may help to explain this heterogeneity in language skills across the autism spectrum (e.g., Groen et al., 2008; Tager-Flusberg, 2018).

### Neural bases of language processing in autism

Various studies have used functional magnetic resonance imaging (fMRI) to investigate the neural bases of language processing in children, adolescents, and adults with autism. Some studies have found that compared to NT individuals, autistic individuals exhibit lower activation, or “brain response,” in bilateral regions of the superior temporal gyrus (STG) and middle temporal gyrus (MTG) when listening to audio recordings of language (Hua et al., 2023; Philip et al., 2012). Other studies have reported that autistic individuals show differences in structural and functional lateralization when compared to NT individuals (Herringshaw, Ammons, DeRamus, & Kana, 2016; Lindell & Hudry, 2013). Many autistic individuals process language in right-lateralized regions of the brain (Herringshaw et al., 2016; Lindell & Hudry, 2013), a finding that is often referred to as “right hemisphere dominance” or “reversed functional lateralization,” given that NT individuals typically process language in left-lateralized regions of the brain (Friederici, 2011). A meta-analysis by Herringshaw and colleagues (2016) found that this right hemisphere dominance in autistic individuals is localized to regions of the right STG and inferior frontal gyrus (IFG).

Group differences in functional lateralization emerge during infancy and become more apparent with age (Morrel, Singapuri, Landa, & Reetzke, 2023). Indeed, language processing becomes more left-lateralized in NT children and more right-lateralized in autistic children during the first few years of life (Eyler, Pierce, & Courchesne, 2012; Flagg, Cardy, Roberts, & Roberts, 2005; Redcay & Courchesne, 2008). Unfortunately, only a handful of fMRI studies have investigated the neural bases of language processing in preschool-aged autistic children (e.g., Eyler, Pierce, & Courchesne, 2012; Lombardo et al., 2015; Redcay & Courchesne, 2008). This gap in the literature is concerning, as the first five years of life are considered to be a sensitive period in language development. Most autistic children who do not acquire spoken language by 5 years of age will continue to remain minimally verbal into adulthood (Mody & Belliveau, 2013; Tager-Flusberg & Kasari, 2013), highlighting the preschool years as developmental period worthy of further research.

As previously mentioned, individual differences in how the brain processes language may help to explain why language skills are so heterogenous across children on the autism spectrum. However, few studies have investigated the association between autistic children’s brain response to language and their language skills. Studies that have examined this association in preschool-aged children with autism have reported a mix of positive (Redcay & Courchesne, 2008), negative (Lombardo et al., 2015), and non-significant (Eyler, Pierce, & Courchesne, 2012) associations between children’s brain response to language and their language skills. Given these inconsistent findings, further research is needed to verify whether this brain-behavior association is present in autistic children during the preschool years.

### Using fMRI to study the brain during social contexts

The biggest limitation of existing research on the neural bases of language processing in both NT and autistic children is that findings may not be generalizable to real-world “social contexts” in which children listen to and interact with a live social partner. In contrast, the majority of fMRI studies to date have presented language stimuli during “non-social contexts” in which children listen to audio recordings of stories and other speech sounds while socially isolated inside of a fMRI scanner. These non-social contexts are in stark contrast to how children are exposed to language in everyday life, which jeopardizes the ecological validity of study findings.

Some researchers have found creative ways to study how the brains of NT children process language during social contexts inside of the fMRI scanner. For example, one fMRI study conducted with NT school-aged children found that the temporal parietal junction (TPJ) responds more strongly to language spoken by a live experimenter compared audio recordings of language (Rice, Moraczewski, & Redcay, 2016; also see Rice & Redcay, 2016 for study conducted with NT adults). While this study established that the brains of NT children process language differently during social and non-social contexts, this study was still conducted inside of a noisy, spatially restrictive fMRI scanner using stimuli that was presented through speakers and a computer screen. Recent research has shown that the brain responds differently to in-person social interactions compared to social interactions over video chat (Zhao et al., 2023), further highlighting the importance of measuring brain function during naturalistic neuroimaging tasks that closely resemble the social contexts that children experience in everyday life.

To our knowledge, no fMRI studies to date have examined how the brains of autistic children process language during social contexts. This gap in the literature is likely due to the methodological limitations of using fMRI with young, clinical populations (Vanderwert & Nelson, 2014). However, a handful of studies have explored how the brains of autistic adults function during social contexts inside of the fMRI scanner. For example, studies have found that autistic adults show lower activation than NT adults in regions of the brain responsible for joint attention and mentalizing (Krall et al., 2015; Mundy, 2018), including the bilateral MTG and right posterior superior temporal sulcus (STS; subregion of the TPJ) during social contexts inside of the fMRI scanner (Assaf et al., 2013; Redcay et al., 2013; Redcay & Saxe, 2013). These differences in brain function may help to explain why autistic individuals experience challenges with language, communication, and social interaction (Rolison, Naples, & McPartland, 2015). Nevertheless, further research is needed to understand how the brains of autistic children process language during social contexts, and to explore whether this brain function relates to children’s language skills.

### Using fNIRS to study the brain during in-person social contexts

Functional near-infrared spectroscopy (fNIRS) is a non-invasive neuroimaging method that accounts for many of the methodological limitations of fMRI. fNIRS is robust to motion artifacts, quiet, and most importantly, it allows researchers to measure children’s brain function while they are awake and interacting in-person with a live social partner (McDonald & Perdue, 2018). fNIRS uses near-infrared light to measure changes in concentration of oxygenated hemoglobin (HbO) and deoxygenated hemoglobin (HbR) on the cortical surface of the brain. Increases in HbO and corresponding decreases in HbR reflect increases in blood volume to brain regions that are experiencing increased neuronal activity (Obrig & Villringer, 2003). Previous work has shown that the hemodynamic response measured by fNIRS is highly correlated with the BOLD signal measured by fMRI, supporting fNIRS as an alternative neuroimaging method to fMRI (Sassaroli et al., 2006).

In recent years, researchers have begun using fNIRS to study how the brains of NT infants, children, and adults function during in-person social contexts (see Gervain, Minagawa, Emberson, & Lloyd-Fox, 2023 and Quaresima & Ferrari, 2019 for review). fNIRS has also been used to study brain function in autistic infants, children, and adults (Conti et al., 2022), although few of these studies utilized naturalistic neuroimaging tasks that closely resemble the social contexts that we experience in everyday life. A handful of more recent fNIRS studies have compared how the brains of NT and autistic individuals function during in-person social contexts, such as mutual eye gaze in adults and turn-taking games in school-aged children (Hirsch et al., 2022; Su, Culotta, Tsuzuki, & Bhat, 2022; Su et al., 2020; Su et al., 2023). These studies reported group differences in the strength and localization of brain response, suggesting that the brains of autistic individuals function differently than the brains of NT individuals during in-person social contexts. However, no studies to date have used fNIRS to study how the brains of preschool-aged NT and autistic children process language during in-person social contexts.

### Book reading as a social context and screen time as a non-social context

Book reading is one social context in which we can study the neural bases of “live language” processing. Book reading is one of the earliest and most common household activities in which children are exposed to language spoken by a live social partner (Hoyne & Egan, 2019). Book reading has also been shown to support language and literacy development in both NT children (Noble et al., 2019) and autistic children (Boyle, McNaughton, & Chapin, 2019). “Screen time,” defined as the use of screen-based media, is one non-social context in which we can study the neural bases of “recorded language” processing. Screen time has become a common and preferred solitary household activity for NT children (Chen & Adler, 2019) and autistic children (Slobodin, Heffler, & Davidovitch, 2019). In contrast to book reading, too much screen time has been linked to lower language skills in both NT children (Madigan et al., 2020) and autistic children (Dong et al., 2021). fNIRS provides us with the unique opportunity to study the neural bases of language processing during book reading and screen time, two household activities that children experience in everyday life.

### Present study

The present study investigated the neural bases of language processing during social context and non-social contexts in a sample of preschool-aged autistic and NT children. Using fNIRS, we measured children’s brain response during two conditions – a live language condition, in which children listened to a story that was spoken by a live experimenter during an in-person social context (i.e., book reading), and a recorded language condition, in which children listened to a story that was played via an audio recording during a non-social context (i.e., screen time).

For our first two aims, we identified which regions of the brain were active during the live language condition and the recorded language condition within each group and compared the strength of brain response during the live language condition to the strength of brain response during the recorded language condition within each group. We hypothesized that NT children would show greater brain response during the live language condition compared to the recorded language condition in the TPJ (Alkire, Levitas, Warnell, & Redcay, 2018; Rice, Moraczewski, & Redcay, 2016; Rice & Redcay, 2016; Warnell, Sadikova, & Redcay, 2018), while autistic children would show similar brain response during both conditions (Assaf et al., 2013; Redcay et al., 2013; Redcay & Saxe, 2013). Our third aim was to determine whether the strength of brain response during each condition differed between groups. We hypothesized that autistic children would show greater right-lateralized activation and lower left-lateralized activation compared to NT children during both conditions in regions of the frontal and temporal gyri (Eyler, Pierce, & Courchesne, 2012; Lombardo et al., 2015; Redcay & Courchesne, 2008). For our fourth aim, we explored the associations between children’s brain response during the live language condition and recorded language condition and their language skills. We predicted that greater brain response during both conditions would be associated with higher language skills (Redcay & Courchesne, 2008; Smith et al., 2020), but we did not have specific hypotheses about which brain regions would show this association.

## Methods

### Participants

Children between 36 and 72 months of age were recruited using a local database of families who expressed interest in contributing to research on child development and/or autism, social media posts and flyers shared in the Greater Boston and New England area (Massachusetts, New Hampshire, Rhode Island, and Connecticut), local intervention centers, and the SPARK research match program (Fombonne et al., 2022). Children were enrolled in either the Autism Spectrum Disorder (ASD) group or the Neurotypical (NT) group. Before enrollment in the present study, interested families consented to an online screening process to confirm their eligibility. To be eligible for the present study, children in the ASD group needed a community diagnosis of Autism Spectrum Disorder from a medical professional, which was confirmed in lab using the second edition of the Autism Diagnostic Observation Schedule (Lord et al., 2012). Children in the NT group had no developmental, behavioral, speech, and/or language disorders and no family history of autism in any first-degree relatives. For both the ASD group and the NT group, children who had a history of seizure disorders, genetic syndromes, severe head injury, or pre-term birth (< 32 weeks) were ineligible to participate in the present study. Because fNIRS stimuli, behavioral assessments, and parent-report measures were presented in English, children in both groups also needed to be from predominately English-speaking households (i.e., heard English > 50% of the time at home). No other exclusion criteria were used.

The final sample included *N* = 20 children in the ASD group (3 female, 17 male) between the ages of 3 years 0 months 22 days and 5 years 11 months 17 days (*M* = 4.30 years old, range = 3.06–5.96 years old) and *N* = 20 age- and sex-matched children in the NT group (5 female, 15 male) between the ages of 3 years 2 months 0 days and 5 years 11 months 28 days (*M* = 4.25 years old, range = 3.17–5.99 years old). Behavioral data and fNIRS data were collected from an additional eight NT participants who were dropped from the current sample after group matching procedures (Bang, Sharda, & Nadig, 2020). Behavioral data were collected from an additional six participants who were excluded from the current sample for the following reasons: no fNIRS data collection because child could not tolerate wearing the fNIRS cap (3 ASD, 1 NT), lost contact between first and second visit (1 ASD), had family history of autism in first-degree relative (1 NT). Demographic information for the final sample by group is provided in Table 1.

### Procedure

Families came to our research center at Boston University for one or two in-person visits, each lasting between two and three hours with breaks. The number of visits and duration of each visit was tailored to the needs of each family. During the first visit, we obtained informed consent from the parent, on behalf of the child, as well as assent from the child, which was facilitated using a social story. Children in both groups then completed behavioral assessments with a trained researcher, including the fifth edition of the Preschool Language Scales (Zimmerman, Steiner, & Pond, 2011). Children in the ASD group also completed the second edition of the Autism Diagnostic Observation Schedule (Lord et al., 2012) with a research reliable administrator to confirm their community diagnosis of ASD. All parents completed a series of parent-report measures, including a demographics questionnaire.

At the end of the first visit, children completed a desensitization procedure to make sure that they were comfortable wearing a practice fNIRS cap (Tager-Flusberg et al., 2017). During the second visit, children completed the fNIRS task described below. Any behavioral assessments that were not completed during the first visit were completed during the second visit, which occurred within one month of the first visit (*M* = 9.10 days, range: 0–41 days). All study procedures were conducted according to the guidelines of the Declaration of Helsinki and approved by the Institutional Review Board at Boston University (protocol #5334).

### fNIRS task

**Figure 1:**
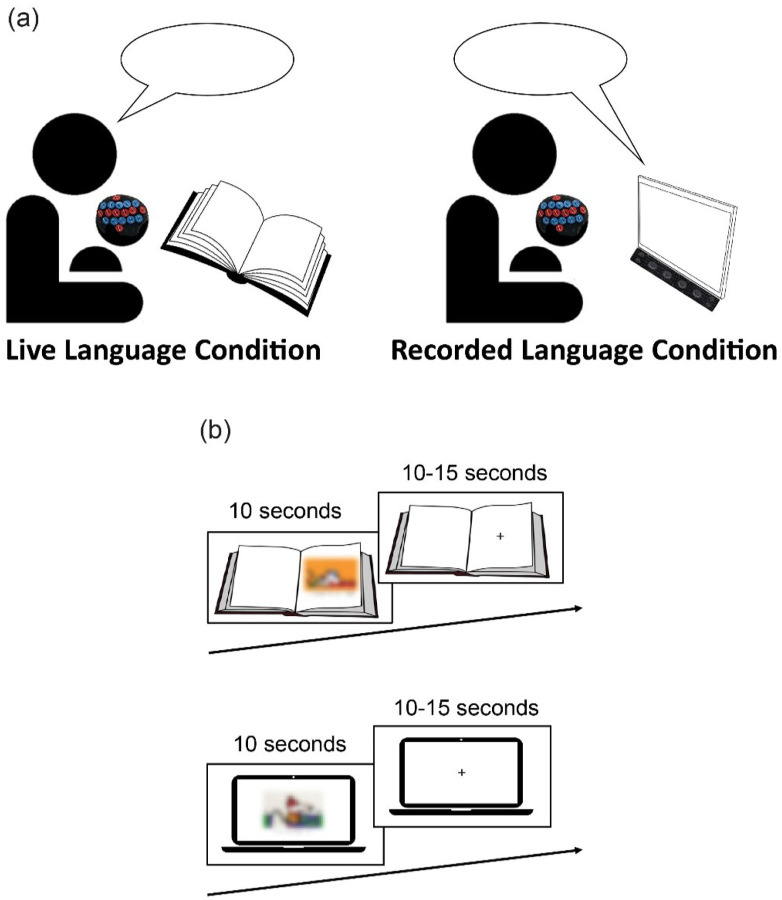
fNIRS task design *Note*. (a) The fNIRS task included two conditions – a live language condition and a recorded language condition. The live language condition was designed to simulate book reading and the recorded language condition was designed to simulate screen time. (b) Depiction of stimulus presentation for the live language condition and the recorded language condition. Each trial was presented for 10 seconds, followed by a jittered fixation cross for 10–15 seconds. Each condition included 18 trials.

For the fNIRS task, each child listened to two different stories. The stories, which were called “Winter” and “Summer,” were created by the study’s first author and reviewed by two speech language pathologists to ensure that they were matched on number of words, syntax, vocabulary, content/sequence of events, and number of characters. One story was presented in the live language condition and the other story was presented during the recorded language condition (Fig. 1a).

In the live language condition, a live female experimenter initiated interaction with the child by reading them one of the scripted stories from a printed book. While listening to the story, the child was seated next to the experimenter so that they could see each page of the book, which was placed on a stand in front of the child. Each page of the book, which served as one trial, included one simple illustration and typed text that matched what the child was hearing. The live language condition was designed to simulate the household activity of book reading. To ensure that consistent prosody and pacing was used across trails in the live language condition, the experimenter shadowed a pre-recorded version of the story that was presented to her through earbud headphones that could not be seen or heard by the participant.

In the recorded language condition, the child listened to an audio recording of the same female experimenter reading the other scripted story. Each trial included one simple illustration and typed text that matched what the child was hearing. Trial visuals were presented using a computer screen instead of a printed book. The computer screen was placed in front of the child and a speaker that played the audio recording was placed below the computer screen. In this condition, the experimenter remained seated next to the child to control for the presence of a social partner, but the experimenter did not initiate interaction with the child during the trials. The recorded language condition was designed to simulate the household activity of screen time/screen-based media use. This condition was also designed after more conventional neuroimaging tasks which use audio recordings and screen-based visual stimuli. Visual and audio stimuli were presented using the E-prime 3.0 software (Psychology Software Tools, 2016).

Each story was divided to create 18 trials that lasted 10 seconds each. Between each trial, a jittered fixation cross was presented for 10 to 15 seconds to ensure that the hemodynamic response returned to baseline before the next trial was manually triggered by the experimenter (Fig. 1b). 18 trials of one condition were presented during the first run, followed by a short break, and then 18 trials of the other condition were presented during the second run. The order of conditions, as well as the story used in each condition (“Winter” or “Summer”), were randomized and counterbalanced across participants so that each child received both conditions and listened to both stories. Each condition lasted between 8 and 10 minutes. The entire fNIRS task lasted no longer than 60 minutes, including time for set up, capping, and breaks.

The child remained seated next to the experimenter in a dimly lit room throughout the fNIRS task. Children were instructed to look and listen to each story while keeping their voice quiet and their body still in order to minimize the presence of motion artifacts during data collection. Snacks or handheld fidget toys were used if the child became restless or attempted to take the fNIRS cap off. After excluding trials in which the experimenter made an administration error (e.g., did not turn page of book at the correct time, deviated from book script), children in the ASD group completed an average of 16.50 trials for the live language condition (range: 4–18) and 17.55 trials for the recorded language condition (range: 11–18). Children in the NT group completed an average of 17.80 trials for the live language condition (range: 16–18) and 18.00 trials for the recorded language condition. The number of trials per condition did not significantly differ between groups (Live language: *t* = 1.439, *p* = .166; Recorded language: *t* = 1.280, *p* = .216).

### fNIRS system and cap

**Figure 2:**
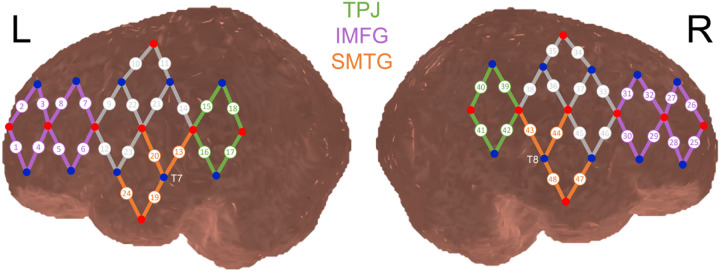
fNIRS probe design projected onto a 4 year old brain template Note. Sources are depicted as red circles and detectors are depicted as blue circles. Channels are depicted as lines between source-detector pairs. The fNIRS probe was designed so that channels bilaterally cover regions of interest in the frontal and temporal lobes, including 8 channels over the inferior and middle frontal gyrus (IMFG), shown in purple, 4 channels over the superior and middle temporal gyrus (SMTG), shown in orange, and 4 channels over the temporal parietal junction (TPJ), shown in green. Detectors 4 and 14 covered the T7 and T8 landmarks, respectively. Channels shown in grey did not cover brain regions of interest and were thus excluded from analyses. Brain template provided by the Neurodevelopmental MRI Database (Richards, Sanchez, Phillips-Meek, & Xie, 2015).

fNIRS data were collected using the TechEn CW7 fNIRS system operating at 690nm and 830nm wavelengths (TechEn Inc) and a 50 Hz sampling frequency. The fNIRS cap included a probe of 16 light sources and 20 detectors to make up 48 channels with a distance of approximately 25mm. The probe was designed using the AtlasViewer toolbox (Aasted et al., 2015) so that detectors 4 and 14 covered the T7 and T8 landmarks, respectively. Channel placement over brain regions of interest (ROIs) was determined by visually inspecting the probe design projected onto a 4 year old brain atlas (Fig. 2), in addition to projecting the probe design onto a resized adult brain atlas that provided the specific MNI coordinates for each channel. Channel placement and ROIs were cross referenced using the devfOLD toolbox (Fu & Richards, 2021). Channels bilaterally covered brain regions of interest (ROIs) in the frontal, temporal, and parietal lobes, including the inferior and middle frontal gyri (IMFG), the superior and middle temporal gyri (SMTG), and the temporal parietal junction (TPJ; including regions of the supramarginal gyrus and angular gyrus). These ROIs were broadly defined as the IMFG, SMTG, and TPJ, given the possibility of slight variation in probe placement across participants. ROIs were selected a priori based on previous studies that have demonstrated that these brain regions are involved in language processing, narrative comprehension, and social cognitive processes (Enge, Friederici, & Skeide, 2020; Horowitz-Kraus & Hutton, 2015; Mar, 2011). Cap sizes of 50cm or 52cm were used, depending on the child’s head circumference.

To guide accurate and symmetrical placement of the fNIRS cap on each child’s head, the distances between naison-inion (Nz-Iz) landmarks and left-right pre-auricular (LPA-RPA) landmarks were measured. These distances were halved to locate the midpoints of Nz-Iz and LPA-RPA so that Cz could be identified and marked. The Cz mark on the fNIRS cap was aligned with Cz on each child’s head during cap placement. The fNIRS cap was secured with a chin strap and a light attenuating shower cap was placed over the fNIRS cap to shield the detectors from possible light interference.

### fNIRS data processing

fNIRS data were processed using Homer3 (v1.81.4; Huppert et al., 2009). First, channels with a poor signal to noise ratio in the raw signal were removed from analyses (*hmrR_PruneChannels*; dRange = 1e3 1e7, SNRthresh = 4; see Supplementary Materials Figure S1 for information about excluded channels per participant). The average percentage of channels retained for analyses did not significantly differ between groups (ASD: 49.06% channels retained; NT: 62.24% channels retained; *t* = 1.450, *p* = .155). The raw signal from remaining channels was converted to optical density (*hmrR_Intensity2OD*) and motion artifact correction was performed using the spline interpolation with Savitzky–Golay filtering method (*hmrR_MotionCorrectSplineSG*; p = 0.99, framesize = 10; Jahani, Setarehdan, Boas, & Yücel, 2018). Next, a low pass filter was used to exclude high frequency fluctuations (*hmrR_BandpassFilt*; lpf = 0.50). Then, optical density was converted into oxygenated hemoglobin (HbO) and deoxygenated hemoglobin (HbR) concentration changes using the Modified Beer-Lambert law (*hmrR_OD2Conc*; ppf = 1); signal changes are presented as the products of concentration changes and mean path length (M*mm; Yücel et al., 2021). Finally, the hemodynamic response function (HRF) was estimated over the time range of −2 to 18 seconds using the general linear model (GLM) with the least-squares method for estimating the weights of consecutive Gaussian functions (*hmrR_GLM*; glmSolveMethod = 1, idxBasis = 1, driftOrder = 3). To remove global systemic physiology from the signal (i.e., physiological noise), we used the average of all channels as a regressor in the GLM (i.e., common average reference method; Klein et al., 2023). After data processing, average HbO and HbR concentration values from 0 to 10 seconds (i.e., trial length) were calculated and used for analyses.

### Measures

#### Preschool Language Scales, Fifth Edition (PLS)

All children completed the fifth edition of the PLS (Zimmerman, Steiner, & Pond, 2011), a comprehensive language assessment that evaluates two domains of language – expressive communication and auditory comprehension. PLS total language standard scores were used as a measure of children’s language skills.

#### Demographics questionnaire

All parents completed a demographics questionnaire that gathered information about children’s age, sex assigned at birth, race, and ethnicity, as well as family characteristics, including language(s) spoken at home, parent education level, and annual household income.

#### Analyses

All analyses were conducted using HbO concentration values and were carried out using SPSS (v 27.0). Supplementary channel analyses are provided in the Supplementary Materials.

#### ROI analyses

For region of interest (ROI) analyses, we calculated average HbO concentration values for each ROI (i.e., “ROI averages”) by averaging HbO concentration values across all channels covering each ROI (see Fig. 2 for specific channel numbers in each ROI). Using these ROI averages, we first conducted within-group one-sample t-tests to identify ROIs that showed significant activation (i.e., ROI averages significantly greater than zero baseline) or deactivation (i.e., ROI averages significantly less than zero baseline) during each condition. Next, we conducted within-group paired sample t-tests to determine whether ROI averages significantly differed during the live language condition compared to the recorded language condition. We then conducted between-group independent samples t-tests to determine whether ROI averages during each condition differed between groups.

#### Correlation analyses

Pearson’s correlation analyses were conducted to explore the associations between children’s brain response and their language skills. More specifically, we examined correlations between ROI averages during the live language condition and the recorded language condition and children’s PLS total language standard scores. We also examined correlations between condition contrasts (absolute value of the difference between ROI averages during the live language condition and ROI averages during the recorded language condition) and children’s PLS total language scores. Correlation analyses were conducted using the entire sample to increase statistical power.

## Results

### Sample demographics and descriptives

When comparing demographic characteristics of children in the ASD group to children in the NT group, groups did not significantly differ on child age (*t* = .151, *p* = .881), sex (χ^2^ = .625, *p* = .429), race (χ^2^ = 4.114, *p* = .249), or ethnicity (χ^2^ = 3.243, *p* = .072), nor did they differ on parent education level (χ^2^ = 8.976, *p* = .062), annual household income (χ^2^ = 5.419, *p* = .712), or language(s) spoken at home (χ^2^ = .102, *p* = .749). PLS total language standard scores (*t* = 4.845, *p* < .001) were significantly lower in the ASD group than the NT group.

### ROI analyses

#### NT group

Within the NT group, HbO concentration values were significantly greater than zero in the right TPJ during the live language condition, indicating significant activation (*t*(17) = 3.078, p = .007, *M*_*diff*_ =.000012, 95% CI: [.000004, .000020]; Cohen’s d = .726, 95% CI: [.196, 1.239]). During the recorded language condition, HbO concentration values were significantly greater than zero in the right SMTG, indicating significant activation (*t*(15) = 3.370, *p* = .004, *M*_*diff*_ = .000021, 95% CI: [.000008, .000034]; Cohen’s d = .843, 95% CI: [.258, 1.406]). HbO concentration values were significantly less than zero in the right IMFG during the recorded language condition, indicating significant deactivation (*t*(18)=−2.815, p = .011, *M*_*dif*f_ = −.000009, 95% CI: [−.000016, − .000002]; Cohen’s d=−.646, 95% CI: [−1.134, − .142]).

**Figure 3:**
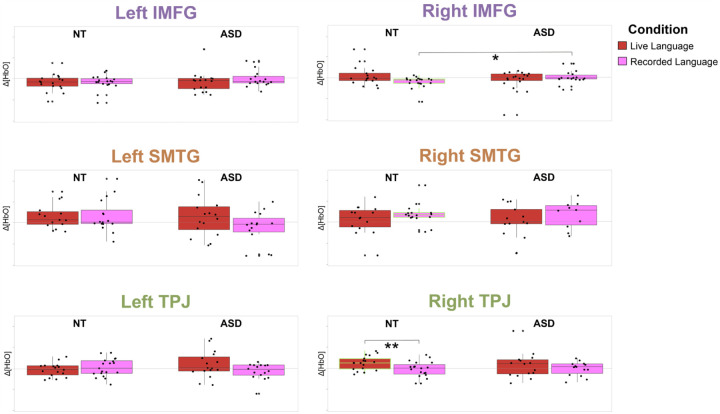
Average HbO concentration values during the live language condition and recorded language condition in all ROIs by group Note. * p<.05, ** p<.01. ROI averages during the live language condition are represented in red. ROI averages during the recorded language condition are represented in magenta. ROI averages outlined in green showed a significant difference in HbO relative to baseline. The y-axis shows a range of HbO concentration values from −9.0×10^−5^ to 11.0×10^−5^ M*mm. Error bars represent 95% CI. ASD=Autism Spectrum Disorder, NT=Neurotypical, ROI=Region of Interest, IMFG=Inferior and Middle Frontal Gyrus, SMTG=Superior and Middle Temporal Gyrus, TPJ=Temporal Parietal Junction

When comparing HbO concentration values between conditions, HbO concentration values were significantly greater during the live language condition than the recorded language condition in the right TPJ (*t*(17) = 3.085, *p* = .007, *M*_*diff*_ = .000014, 95% CI: [.000005, .000024]; Cohen’s d = .727, 95% CI: [.197, 1.240]; Fig. 3). In all other ROIs, there were no significant differences in HbO concentration values between conditions (Table 2).

Within the ASD group, no ROIs showed evidence of significant activation or deactivation during either condition (i.e., HbO concentration values did not significantly differ from zero; *ps* ≥ .099). When comparing HbO concentration values between conditions, HbO concentration values did not significantly differ between conditions in all ROIs (Table 2; Fig. 3).

#### NT group vs ASD group

During the live language condition, HbO concentration values did not significantly differ between groups in all ROIs (Table 2). During the recorded language condition, HbO concentration values were significantly lower in the NT group than the ASD group in the right IMFG (*t*(35)=−2.495, *p* = .017, *M*_*diff*_ = − .000012, 95% CI: [−.000022, − .000002]; Cohen’s d=−.821, 95% CI: [−1.488, − .145]; Fig. 3). In all other ROIs, there were no significant differences in HbO concentration values between groups (Table 2).

#### Correlation analyses

**Figure 4:**
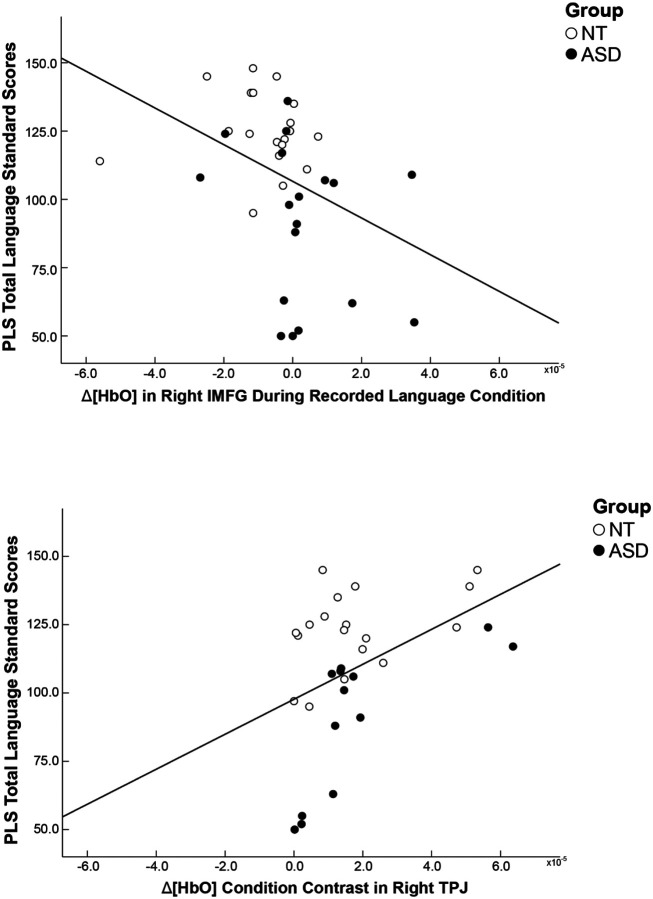
Correlations between children’s brain response to language and their language skills Note. Correlation analyses were conducted using the entire sample. ASD=Autism Spectrum Disorder, NT=Neurotypical, ROI=Region of Interest, IMFG=Inferior and Middle Frontal Gyrus, TPJ=Temporal Parietal Junction, PLS=Preschool Language Scales

Across both groups, PLS total language scores were not significantly correlated with HbO concentration values during the live language condition in any of the ROIs (*ps* ≥ .304). PLS total language scores were significantly and negatively correlated with HbO concentration values during the recorded language condition in the right IMFG (*r*(35)=−.373, *p* = .023), indicating that children who had lower language skills showed greater brain response to recorded language in the right IMFG (Fig. 4).

When examining correlations between PLS total language scores and condition contrasts (absolute value of the difference between HbO concentration values during the live language condition and HbO concentration values during the recorded language condition), PLS total language scores were significantly and positively correlated with difference in brain response between conditions in the right TPJ (*r*(29) = .436, *p* = .014), indicating that children who had higher language skills showed a greater difference in brain response to live language compared to recorded language in the right TPJ (Fig. 4). PLS total language scores were not significantly correlated with condition contrasts in any of the other ROIs (*ps* ≥ .235).

## Discussion

The present study investigated the neural bases of language processing during social and non-social contexts in preschool-aged autistic and NT children. Using fNIRS, we measured children’s brain response to live language presented during an in-person social context (i.e., book reading) and recorded language presented during a non-social context (i.e., screen time). Analyses revealed that in the NT group, the right TPJ was active during the live language condition and the right SMTG was active during the recorded language condition. Also, brain response during the live language condition was greater than brain response during the recorded language condition in the right TPJ. In the ASD group, no brain regions showed evidence of significant activation during either condition, and the strength of brain response did not significantly differ between conditions. When comparing the strength of brain response between groups, the ASD group showed greater brain response during the recorded language condition in the right IMFG compared to the NT group. There were no significant group differences in the strength of brain response during the live language condition. Correlation analyses demonstrated that across groups, children’s language skills were negatively associated with brain response to recorded language in the right IMFG. Children’s language skills were also positively associated with the difference in brain response to live language versus recorded language in the right TPJ. Taken together, these findings indicate that the brains of NT children, but not autistic children, process language differently depending on whether it’s presented during a social context or a non-social context. Individual differences in how the brain processes language during social and non-social contexts may help to explain why language skills vary across autistic and NT children.

### Greater right TPJ brain response during the live language condition than the recorded language condition in the NT group only

In the NT group, the right TPJ was active during the live language condition only. Additionally, NT children’s brain response in the right TPJ was greater during the live language condition than the recorded language condition. Together these findings suggest that for NT children, the right TPJ is involved in processing live language during social contexts, such as book reading, but not involved in processing recorded language during non-social contexts, such as screen time. These findings align with those reported in Rice, Moraczewski, and Redcay (2016), who found that the brains of NT school-aged children responded more strongly to live language than recorded language in the right TPJ, as well as other studies of NT school-aged children that have documented right TPJ activation during social contexts inside of the fMRI scanner (Alkire et al., 2018; Warnell, Sadikova, & Redcay, 2018).

The right TPJ is involved in a variety of social cognitive processes, including joint attention (Mundy, 2018; Saxe, 2010) and mentalizing (Frith & Frith, 2003; Krall et al., 2015). fNIRS studies conducted with NT infants, children, and adults have documented right TPJ activation during in-person joint attention tasks (Dravida et al., 2020; Hakuno et al., 2018) and computer-based mentalizing tasks (e.g., false belief tasks; Hyde et al., 2018; Wysocka et al., 2020). Given the role that the right TPJ plays in these social cognitive processes, our findings suggest that NT children may have been engaged in joint attention and/or mentalizing to a greater extent during the live language condition than the recorded language condition. It may also be that the right TPJ is sensitive to distinguishing between social and non-social contexts.

Indeed, researchers have suggested that simply having the opportunity to interact with a live social partner can elicit activation in the right TPJ (Redcay et al., 2010). Our findings indicate that listening to language spoken by a live social partner engages the right TPJ in NT preschool-aged children, even in the absence of explicit bids for joint attention or prompts for mentalizing.

In contrast to what was found in the NT group, the right TPJ did not show evidence of significant activation during the live language condition in the ASD group. Furthermore, the strength of brain response did not significantly differ between conditions in the ASD group in any regions, indicating that the brains of autistic children respond similarly to live language presented during a social context and recorded language presented during a non-social context. Previous studies have also found that the brains of autistic individuals function similarly during social and non-social contexts. For example, Redcay and colleagues (2013) found that NT adults showed greater brain response during a computer-based interactive joint attention task compared to a computer-based non-interactive solo attention task in the right posterior STS, a subregion of the TPJ, while autistic adults showed equivalent brain response during both tasks. Similar findings were reported in a study conducted with autistic adolescents (Oberwelland et al., 2017; but see Redcay & Saxe, 2013). Other studies found that NT adults exhibited greater right TPJ activation during mentalizing tasks compared to non-mentalizing tasks, while autistic adults did not show differences in right TPJ activation between tasks (Lombardo et al., 2011; Mason et al., 2008). Taken together, these findings suggest that autistic individuals show similar brain response in the right TPJ during social and non-social contexts.

There are several explanations for why we didn’t observe evidence of significant right TPJ activation in the ASD group during the live language condition. First, the right TPJ may not be functionally specialized to process socially relevant information (e.g., mental states of a social partner) in children with autism. It is possible that a different region of the brain, besides the right TPJ, is responsible for processing social relevant information in autistic children (e.g., right IFG; Redcay et al., 2010; Yuk et al., 2018), although this was not observed in the present study. It may also be that children in the ASD group were not attending to socially relevant information during the live language condition, as recent research has demonstrated that autistic children show reduced attention to socially salient stimuli (e.g., face of the reader) during book reading (Ambarchi et al., 2024). Another possible explanation for why we did not observe right TPJ activation in the ASD group is that autistic children may not have been engaged in joint attention or mentalizing during the live language condition. It is common for autistic individuals to experience challenges engaging in joint attention (Bottema-Beutel, 2016) and mentalizing (Chung, Barch, & Strube, 2014), especially during book reading (Rumpf, Kamp-Becker, Becker, & Kauschke, 2012; Wicks, Westerveld, Stainer, & Paynter, 2022). Future studies should investigate whether activation in the right TPJ during in-person social contexts relates to children’s joint attention and mentalizing abilities. Additionally, future studies should continue to explore which regions of the brain are responsible for processing socially relevant information during in-person social contexts in autistic individuals.

### Right SMTG activation and right IMFG deactivation during the recorded language condition in the NT group only

ROI analyses revealed evidence of significant activation in the right SMTG during the recorded language condition in the NT group only. Contrary to our hypotheses, we did not observe evidence of significant activation in any left-lateralized brain regions during either condition. This finding was somewhat surprising, given that previous fMRI studies have found that NT children process language in the bilateral temporal gyri (.g., Enge, Friederici, & Skeide, 2020). Nevertheless, this right-lateralized activation could be a result of the stimuli used in the present study. Our task included auditory narratives, which have been shown to elicit activation in primarily right-lateralized regions of the brain (Lindell, 2006; Vigneau et al., 2011). For example, one fMRI study conducted with NT school-aged children found significant activation in the right STG, but not the left STG, during an auditory narrative comprehension task (Schmithorst, Holland, & Plante, 2006). Additionally, our task included both audio and visual stimuli (i.e., auditory narratives paired with illustrations and text), as opposed to audio only stimuli. This could also help to explain why we observed right-lateralized activation in the present study, as the right temporal gyrus is involved in audiovisual integration (Chen & Spence, 2017). A recent meta-analysis reported that tasks that require participants to pay attention to both audio and visual stimuli elicit activation in the right STG and MTG, while tasks with audio only stimuli elicit activation in the left STG (Gao et al., 2023). The right temporal gyrus is responsible for the audiovisual integration of speech sounds with letters and words during reading as well (Vigneau et al., 2011). For example, Berl and colleagues (2010) found that reading comprehension and listening comprehension tasks both elicited right STG activation in NT school-aged children. Regardless of the reason why we observed this right-lateralized activation, our findings indicate that for NT preschool-aged children, the right SMTG is involved in processing recorded language during non-social contexts.

The NT group also showed evidence of significant deactivation in the right IMFG during the recorded language condition. fNIRS studies of NT infants have similarly observed deactivation in frontal regions of the brain when listening to recorded language (e.g., infant directed speech; McDonald et al., 2019). This deactivation may be the result of blood flow moving away from anterior regions of the brain towards more posterior regions of the brain that did show evidence of significant activation during the recorded language condition (e.g. right SMTG). Alternatively, deactivation of this frontal region may reflect changes in the default mode network, a baseline pattern of brain function that is attenuated during tasks that require attentional control and other goal-directed behaviors (Raichle et al., 2001). Deactivation in the right IMFG may therefore be the result of NT children focusing their attention on the audio and visual stimuli presented during the recorded language condition.

### Greater right IMFG brain response in the ASD group than the NT group during the recorded language condition

When comparing brain response between groups, the ASD group showed greater right IMFG brain response than the NT group during the recorded language condition. In contrast, the NT group showed greater left SMTG brain response than the ASD group during the recorded language condition, although this difference was only marginally significant. Together these findings support the well-established body of literature documenting reversed functional lateralization and right hemisphere dominance in autistic individuals during language processing.

Few fNIRS studies have investigated functional laterality and language processing in autism, and the findings across these studies are mixed (Conti et al., 2022; Doi & Shinohara, 2017). However, several fMRI studies have reported that autistic individuals demonstrate right hemisphere dominance during language processing (Herringshaw et al., 2016; Lindell & Hudry, 2013). In line with our findings, a meta-analysis by Herringshaw and colleagues (2016) found that autistic individuals exhibited greater right IFG activation than NT individuals when listening to audio recordings of language. There is also evidence to suggest that this right hemisphere dominance is present in preschool-aged and school-aged children with autism. For example, fMRI studies have documented greater right IFG and MFG brain response in autistic children than NT children during language processing (Redcay & Courchesne, 2008; Wang, Lee, Sigman, & Dapretto, 2006). Similar findings have been reported in infants at elevated likelihood/increased familial risk for autism, suggesting that these differences in laterality are present during the first year of life (see Morrel et al., 2023 for review). Given that right hemisphere dominance for language processing has also been found in children with other developmental disabilities, such as developmental language disorder (Mayes, Reilly, & Morgan, 2015), hyperactivation of right-lateralized brain regions and/or hypoactivation of left-lateralized brain regions may be contributing to language challenges in autism. Indeed, studies have shown that autistic individuals with co-occurring language delay have greater right hemisphere dominance than autistic individuals without co-occurring language delay (Floris et al., 2021).

Contrary to our hypotheses, the strength of brain response during the live language condition did not significantly differ between groups in any of the ROIs. This finding contrasts with previous fMRI and fNIRS studies of school-aged children and adults that reported that autistic individuals showed lower brain response in the bilateral MTG and STG, IFG and MFG, posterior STS, supramarginal gyrus, and angular gyrus than NT individuals during social contexts (Assaf et al., 2013; Hirsch et al., 2022; Redcay et al., 2013; Redcay & Saxe, 2013; Su et al., 2020; Su et al., 2022; Su et al., 2023). There are several possible explanations for why our findings differed from those reported in previous studies. One possibility is that this group difference in brain response during social contexts is not present in autistic preschool-aged children, but emerges later in development. Another possibility is that this group difference may only appear during less structured social contexts that allow for conversation between social partners. At the risk of reducing experimental control, future studies should use fNIRS to measure autistic children’s brain function during less structured, less predictable, and more naturalistic dyadic social interactions that allow for linguistic exchange between social partners.

### Associations between children’s brain response to language and their language skills

Correlation analyses revealed that across groups, children who demonstrated greater brain response in the right IMFG during the recorded language condition had lower language skills, as measured by the PLS. Assuming that greater brain response in the frontal gyrus reflects increased cognitive effort, this finding suggests that children whose brains have to work “harder” to process recorded language have lower language skills, while children whose brains do not have to work as hard to process recorded language have higher language skills. This finding further supports the argument that hyperactivation of the right IMFG during language processing may contribute to language challenges in children with autism.

This negative brain-behavior association differs from previous fMRI studies of autistic and NT children that have documented positive associations between children’s brain response to audio recordings of language in the right IFG and bilateral STG and MTG and their language skills (Redcay & Courchesne, 2008; Skeide, Brauer, & Friederici, 2016; Wang et al., 2006; but see for Eyler, Pierce, & Courchesne, 2012 nonsignificant associations). Findings do however align with Lombardo and colleagues (2015), who reported negative associations between autistic children’s brain response to audio recordings of language in regions of the bilateral frontal and temporal gyri and their language skills (although this study also found that this association was positive in NT children; Lombardo et al., 2015). These seemingly inconsistent findings across studies could be due to differences in the ages of the samples studied, or the language stimuli/task, language assessments, neuroimaging methods, and statistical methods used. To further clarify whether this association between autistic children’s brain response to language and their language skills is positive or negative, future studies should utilize much larger sample sizes, as brain-behavior associations may be unstable in smaller sample sizes (i.e., under 80 participants; Grady et al., 2021).

Contrary to our hypotheses, children’s brain response during the live language condition was not significantly associated with their language skills. However, we did find that children who showed a greater difference in brain response to live language compared to recorded language in the right TPJ had higher language skills, as measured by the PLS. This finding suggests that children whose brains are “better” at neurally distinguishing between live language and recorded language have higher language skills, while children whose brains are not neurally distinguishing between live language and recorded language have lower language skills. Given the role that the right TPJ plays in social cognitive processes like joint attention and mentalizing (Frith & Frith, 2003; Krall et al., 2015; Mundy, 2018; Saxe, 2010), this finding may indicate that children who were engaged in these social cognitive processes to a greater extent during the live language condition than the recorded language condition have higher language skills. Regularly engaging in joint attention and mentalizing during social contexts in everyday life may strengthen children’s language development overtime. In support of this interpretation, both joint attention and mentalizing skills have been positively linked to language skills in children with and without autism (Bottema-Beutel, 2016; Miller, 2006), and interventions that target these social cognitive skills have been shown to lead to long-term improvements in autistic children’s language skills (e.g., Kasari et al., 2012).

In all, findings from correlation analyses suggest that individual differences in how the brain processes language during social and non-social contexts may help to explain why language skills are so variable across children with and without autism. Future studies should continue to explore whether autistic children’s brain function during various types of in-person social contexts is related to their language skills, as this will provide further insight into the neural bases of language challenges in autism.

### Limitations

The present study had some limitations worth mentioning here. First, some statistical analyses may have been underpowered given that not all participants had useable fNIRS data in all ROIs. This was particularly true for the bilateral SMTG, as several participants had poor fNIRS signal quality in channels covering this region. Given this limitation, findings from the present study should be considered preliminary until they can be replicated in a larger sample. To improve the collection of higher quality data from a larger number of autistic participants, future studies should consider how fNIRS caps and optodes can be adapted to accommodate sensory sensitivities, as well as diverse hair colors and textures. Second, while the present study provides insight into the neural bases of language processing during book reading as one specific social context, the neural bases of language processing likely vary depending on the social contexts. Future studies should investigate the neural bases of language processing in autistic children during less structured and less predictable social contexts with varying cognitive, linguistic, and social demands. Third, while we can make inferences about the social cognitive processes that occurred during the live language condition (e.g., joint attention, mentalizing), we cannot say for certain whether or not children were engaged in these processes during our task. Localizer tasks should be used in future work to identify participant-specific networks involved in joint attention and mentalizing (Fedorenko et al., 2010; Saxe & Kanwisher, 2013) to improve our understanding of how the brains of autistic and NT children process language during social contexts.

## Conclusions

Despite these limitations, the novelty of the present study contributes to the literature in several ways. This pioneering study has documented that it is feasible to measure how the brains of preschool-aged autistic children, an understudied age group, function during an in-person social context. We hope that this study will encourage future researchers to use fNIRS to assess how the brains of young children with autism function during other types of in-person social contexts. Additionally, the present study has important implications for the types of tasks that researchers use to study the neural bases of language processing in young children, and advocates for the use of more naturalistic, ecologically valid neuroimaging tasks in future studies. Lastly, this study has established a link between children’s brain function and their language skills, providing a possible neurobiological explanation for why language skills are so variable across the autism spectrum.

## Figures and Tables

**Table 1:** Sample demographics and descriptives by group

ASD		NT	*p*-value
**Child age (years)**			.881
*Mean (SD)*	4.30 (1.03)	4.25 (.90)	
*Range*	3.06–5.96	3.17–5.99	
**PLS total language standard scores**			< .001
*Mean (SD)*	88.70 (28.71)	123.85 (15.11)	
*Range*	50.00–136.00	95.00–148.00	
**Child sex**			.429
Female	15.00%	25.00%	
Male	85.00%	75.00%	
**Child race**			.249
Asian	25.00%	10.00%	
Black or African American	10.00%	0.00%	
More than one race	20.00%	30.00%	
White	45.00%	60.00%	
**Child ethnicity**			.072
Hispanic or Latino	15.00%	0.00%	
Not Hispanic or Latino	85.00%	100.00%	
**Parent education level**			.062
Some high school	5.00%	0.00%	
High school degree or GED	0.00%	0.00%	
Some college or Associate’s degree	25.00%	0.00%	
Bachelor’s degree	25.00%	15.00%	
Master’s degree	20.00%	40.00%	
Doctorate or professional degree	25.00%	45.00%	
**Annual household income**			.712
$20k-$40k	5.00%	0.00%	
$40k-$60k	5.00%	5.00%	
$60k-$80k	5.00%	5.00%	
$80k-$100k	20.00%	5.00%	
$100k-$120k	15.00%	15.00%	
$120k-$140k	5.00%	0.00%	
$140k-$160k	0.00%	0.00%	
$160k-$180k	5.00%	10.00%	
$180k-$200k	5.00%	15.00%	
More than $200k	30.00%	40.00%	
Prefer not to answer	5.00%	5.00%	
**Language(s) spoken at home**			.749
English only	55.00%	60.00%	
English and another language	45.00%	40.00%	

*Note*. Parent education level reflects the highest education level between both parents/caregivers living in the household. While all participants were from predominately English-speaking households (i.e., heard English > 50% of the time at home), some participants were from multilingual households in which they also heard one or more non-English languages at home (i.e., Spanish, Portuguese, Hindi, Farsi, Thai, Mandarin, Cantonese, Hainanese, Japanese, Italian, Bulgarian, and/or German). *p*-values represent results from chi-square tests and independent samples t-tests. ASD = Autism Spectrum Disorder, NT = Neurotypical, PLS = Preschool Language Scales

**Table 2:** Within-group and between-group comparisons of HbO concentration values in all ROIs

ROI	*p*-value					
NT						
	*Live Language vs. Baseline*		*Recorded Language vs. Baseline*		*Live Language vs. Recorded Language*	
Left IMFG	.083		.075		.851	
Left SMTG	.467		.102		.363	
Left TPJ	.276		.668		.127	
Right IMFG	.460		.011*	(−)	.089	
Right SMTG	.422		.004**	(+)	.213	
Right TPJ	.007**	(+)	.915		.007**	LL > RL
ASD						
	*Live Language vs. Baseline*		*Recorded Language vs. Baseline*		*Live Language vs. Recorded Language*	
Left IMFG	.198		.833		.314	
Left SMTG	.678		.325		.482	
Left TPJ	.285		.172		.093	
Right IMFG	.360		.416		.171	
Right SMTG	.633		.099		.292	
Right TPJ	.414		.855		.203	
ASD vs. NT						
	*Live Language*			*Recorded Language*		
Left IMFG	.893			.181		
Left SMTG	.962			.062		
Left TPJ	.150			.205		
Right IMFG	.238			.017*		ASD > NT
Right SMTG	.870			.861		
Right TPJ	.520			.839		
**ASD group**						

Note.

* *p* < .05,

** *p* < .01. (+) represents a significant increase in HbO relative to baseline (i.e., activation) and (−) represents a significant decrease in HbO relative to baseline (i.e., deactivation). ASD = Autism Spectrum Disorder, NT = Neurotypical, LL = Live Language, RL = Recorded Language, ROI = Region of Interest, IMFG = Inferior and Middle Frontal Gyrus, SMTG = Superior and Middle Temporal Gyrus, TPJ = Temporal Parietal Junction

## Data Availability

The datasets generated and analyzed during the current study are not publicly available due to privacy or ethical restrictions, but are available from the corresponding author on reasonable request.

## List of abbreviations

ADOS: Autism Diagnostic Observation Schedule
ASD: Autism Spectrum Disorder
fMRI: Functional Magnetic Resonance Imaging
fNIRS: Functional Near-Infrared Spectroscopy
GLM: General Linear Model
HbO: Oxygenated Hemoglobin
HbR: Deoxygenated Hemoglobin
HRF: Hemodynamic Response Function
IFG: Inferior Frontal Gyrus
IMFG: Inferior Frontal Gyrus and Middle Frontal Gyrus
MFG: Middle Frontal Gyrus
MTG: Middle Temporal Gyrus
NT: Neurotypical
PLS: Preschool Language Scales
ROI: Region of Interest
SES: Socioeconomic Status
SMTG: Superior Temporal Gyrus and Middle Temporal Gyrus
STG: Superior Temporal Gyrus
STS: Superior Temporal Sulcus
TPJ: Temporal Parietal Junction
